# Differentiation Induction as a Response to Irradiation in Neural Stem Cells In Vitro

**DOI:** 10.3390/cancers11070913

**Published:** 2019-06-29

**Authors:** Jana Konířová, Lukáš Cupal, Šárka Jarošová, Anna Michaelidesová, Jana Vachelová, Marie Davídková, Petr Bartůněk, Martina Zíková

**Affiliations:** 1 Laboratory of Cell Differentiation, Institute of Molecular Genetics of the Czech Academy of Sciences, v. v. i., Vídeňská 1083, 142 20 Prague 4, Czech Republic; 2 Department of Radiation Dosimetry, Nuclear Physics Institute of the Czech Academy of Sciences, v. v. i., Husinec 130, 250 68 Řež, Czech Republic; 3 Department of Dosimetry and Application of Ionizing Radiation, Faculty of Nuclear Sciences and Physical Engineering, Czech Technical University, Břehová 7, 115 19 Prague 1, Czech Republic

**Keywords:** neural stem cells, ionizing radiation, proliferation, proliferative arrest, differentiation, apoptosis

## Abstract

Radiotherapy plays a significant role in brain cancer treatment; however, the use of this therapy is often accompanied by neurocognitive decline that is, at least partially, a consequence of radiation-induced damage to neural stem cell populations. Our findings describe features that define the response of neural stem cells (NSCs) to ionizing radiation. We investigated the effects of irradiation on neural stem cells isolated from the ventricular-subventricular zone of mouse brain and cultivated in vitro. Our findings describe the increased transcriptional activity of p53 targets and proliferative arrest after irradiation. Moreover, we show that most cells do not undergo apoptosis after irradiation but rather cease proliferation and start a differentiation program. Induction of differentiation and the demonstrated potential of irradiated cells to differentiate into neurons may represent a mechanism whereby damaged NSCs eliminate potentially hazardous cells and circumvent the debilitating consequences of cumulative DNA damage.

## 1. Introduction

Cranial irradiation is a useful tool for the treatment of primary and metastatic brain tumors in adult and pediatric patients. The worldwide estimated incidence of central nervous system (CNS) malignancies in children is the second most common after leukemia [1] and intracranial or brain metastases occur in approximately 30% of all cancer patients [2]. Radiotherapy improves the lives of cancer patients; however, the use of this therapy is not without side effects. Brain radiation is associated with neurocognitive decline and reduced performance on neuropsychological testing, especially in children [3,4,5]. Although the effect of radiation-induced damage is multifactorial, it is believed that damage to neural stem cell populations is crucial for the pathogenesis of radiation-induced cognitive dysfunction [6,7].

In the adult mammalian brain, populations of neural stem cells (NSCs) represent the critical reservoir of regenerative cells. NSCs are undifferentiated cells that are defined by their replicative potential and their ability to differentiate into multiple neuronal and glial cell types. The adult brain contains two NSC pools located in the subventricular zone of the lateral ventricles (V-SVZ) [8] and the dentate gyrus of the hippocampus [9]. NSCs represent an extremely diverse population of cells, recognizable mainly by their state of quiescence or activation. Recent advances in single-cell transcriptomics provide useful information about the different states of NSCs, and using multiple molecular markers allows isolation of distinct cell subpopulations. Purification of V-SZV NSCs from the mouse brain revealed four types of cells: dormant NSCs, quiescent NSCs (qNSCs), activated NSCs (aNSCs), and progenitor cells (NPCs). Most NSCs are qNSCs that express prominin and astrocytic marker GFAP. These cells give rise to activated, cycling, and EGFR-positive aNSCs, which differentiate into highly proliferative NPCs and finally to neuroblasts that subsequently migrate through the rostral migratory stream to the olfactory bulb, where they differentiate into mature neurons [10,11,12].

The cytotoxicity caused by ionizing radiation is mainly the result of DNA damage. Radiation induces several forms of DNA damage, which include single-strand breaks, double-strand breaks, sugar and base modification, and DNA–protein crosslinking [13]. In response to DNA damage, cell cycle checkpoints become activated to block cell cycle progression, allowing cells to repair the damage [14]. If the damage is irreversible, apoptosis (programmed cell death) is triggered to eliminate the injured cells. Radiation therapy reduces adult neurogenesis through two mechanisms. Ionizing radiation, by inducing acute apoptosis in dividing cells, mainly aNSCs and NPCs, reduces the pool of mitotic NSCs and, consequently, reduces generation of new neurons [15]. However, at moderate doses of irradiation, proliferation in the neurogenic niches restarts a few days after exposure by recruiting qNSCs [16,17]. Another mechanism that affects neurogenesis after radiation exposure are changes within the NSC microenvironment. The exposure to high doses causes permanent inhibition of NSC proliferation in the neurogenic niche [18] that is a direct consequence of the alteration of the NSC niche [19,20]. Even if NSCs survive irradiation and thus are able to reconstitute neurogenesis, such regeneration may be prevented by inflammation and vascular damage in the stem cell niche [19]. Moreover, it was observed that in irradiated mice, the production of transforming growth factor β1 (TGF-β1) by endothelial cells in the stem cell niche is elevated. The increased synthesis of TGF-β1 supports qNSC dormancy and increases susceptibility of proliferative NSCs to apoptosis [20]. In addition, irradiation may also lead to premature differentiation of neural precursors and adoption of glial fate [19,21].

## 2. Results

### 2.1. NSC Culture Characterization

**Figure 1:**
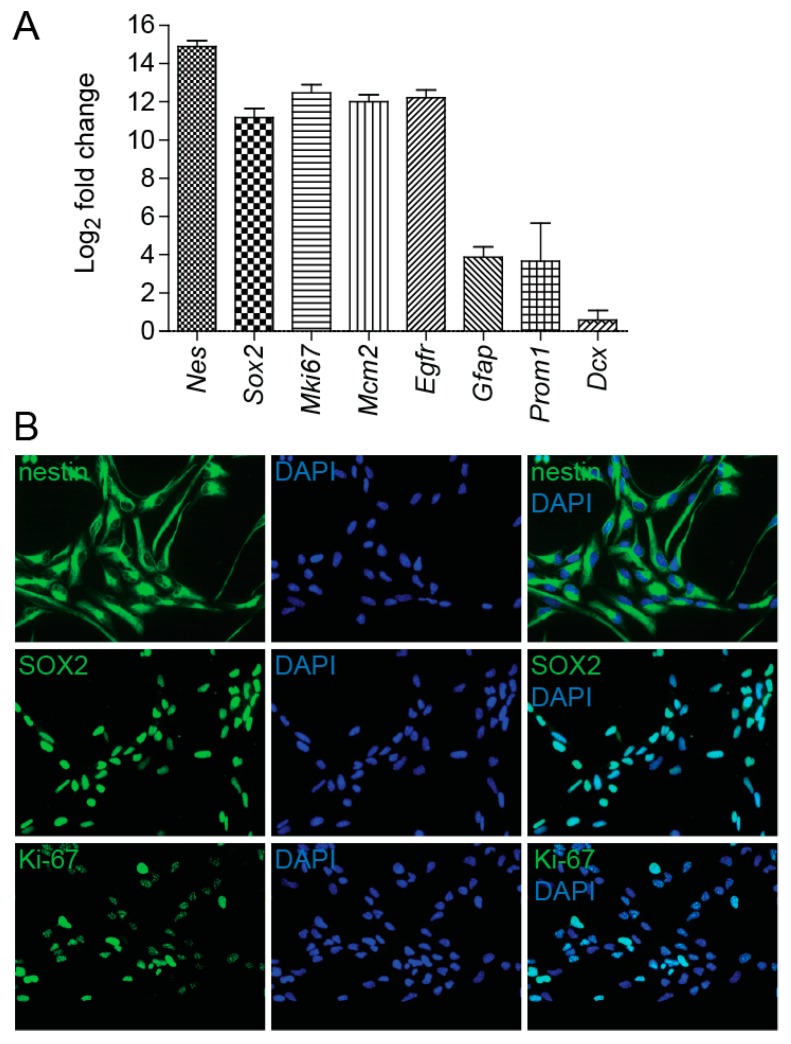
Neural stem cell (NSC) culture characterization. (**A**) Quantitative RT-PCR analysis of mRNA levels of marker genes in NSCs cultured in growth medium in vitro, expressed as Log_2_ fold change. *Gapdh* was used as a reference gene. (**B**) Immunofluorescence images of NSCs stained with nestin, SOX2, and Ki-67 antibodies (green). DAPI (blue) was used to stain the nuclei. Original magnification: 400×.

Our study is focused on evaluating the responses of NSCs to irradiation. To analyze the characteristics of NSCs cultured in vitro we used qRT-PCR and immunocytochemical analysis. Under the specified culture conditions, NSCs were found to express high mRNA levels of stemness markers nestin and *Sox2*. QRT-PCR analysis also showed high expression of *Mki67* and *Mcm2*, markers associated with cell proliferation, and *Egfr*, the established marker for aNSC and NPC subpopulations. We also determined mRNA levels of prominin and *Gfap*, markers of qNSCs and aNSCs, and mRNA levels of *Dcx*, gene exclusively expressed in NPCs and neuroblasts. While cells cultured in growth medium expressed clear levels of prominin and *Gfap*, the expression levels of *Dcx* were almost undetectable (Figure 1A). These results were further confirmed by immunofluorescence studies, which demonstrated nestin, SOX2, and Ki-67 protein expression in cultured cells (Figure 1B).

Using ImageJ software (version 1.48, NIH, Bethesda, MD, USA), we further determined the percentage of Ki-67-positive cells and found that Ki-67 antigen is detectable in 78% of all cells. Together, these results indicate that most cells in culture have features of aNSC late state characterized by high expression of proliferation markers, lower expression of astrocytic markers, and undetectable expression levels of the *Dcx* gene [22]. This is further supported by the proportion of cells negative for Ki-67 antigen (22%), which is considerably greater than the estimated proportion of Ki-67-negative cells (less than 15%) found in NPC populations that were analyzed immediately after the isolation from the mouse brain [22].

### 2.2. In NSCs, Irradiation Induces DNA Damage Response

**Figure 2:**
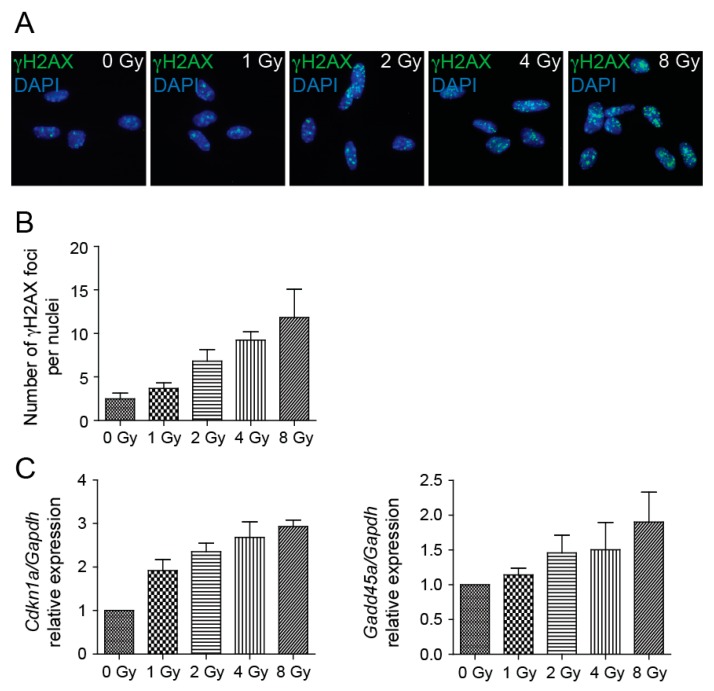
DNA damage response is induced by irradiation. (**A**) Representative immunofluorescence images of NSCs 4 h after irradiation to 0, 1, 2, 4, and 8 Gy doses stained with γ-H2AX antibody (green). DAPI (blue) was used to stain the nuclei. Original magnification: 400×. (**B**) Quantification of γ-H2AX nuclear foci 4 h after irradiation to 0, 1, 2, 4, and 8 Gy doses. Mean values: 0 Gy-2.49, 1 Gy-3.67, 2 Gy-6.82, 4 Gy-9.24, 8 Gy-11.82; *p* = 0.0002. (**C**) Quantitative RT-PCR analysis of mRNA levels of *Cdkn1a* and *Gadd45a* genes 4 h after irradiation to 0, 1, 2, 4, and 8 Gy doses. *Gapdh* was used as a reference gene. Mean values-*Cdkn1a*: 0 Gy-1, 1 Gy-1.92, 2 Gy-2.35, 4 Gy-2.68, 8 Gy-2.93; *p* < 0.0001; -*Gadd45*: 0 Gy-1, 1 Gy-1.14, 2 Gy-1.46, 4 Gy-1.50, 8 Gy-1.9; *p* = 0.0199.

Irradiation of cells produces DNA double-strand breaks (DSBs), and to survive, cells must be able to remove these lesions. To assess DNA damage after NSCs irradiation to 1, 2, 4, and 8 Gy doses, we employed immunofluorescence of γ-H2AX foci. We used an antibody raised to the phosphorylated C-terminal peptide of H2AX and counted the numbers of γ-H2AX nuclear foci. Compared to sham-irradiated control, cultures of NSC showed bright γ-H2AX foci 4 h after irradiation (Figure 2A), the numbers of which increased by increasing doses of radiation (Figure 2B). The cellular response to radiation is complicated and involves activities of many genes, some of which are p53-mediated. The p53 protein is present at higher levels in NSCs than in other cells of the adult mouse brain and acts as a negative regulator of NSCs self-renewal [23]. We determined transcriptional activity of p53 targets cyclin-dependent kinase inhibitor 1A (*Cdkn1a*) and growth arrest and DNA-damage-inducible 45 alpha (*Gadd45a*). When DNA damage is present before the entry into S phase, p53 halts the cell cycle at the G_1_ phase, in part by transcriptionally inducing *Cdkn1a*, also known as p21 [24]. Activated p53 induces *Gadd45a* mRNA, which, when overexpressed, is sufficient to induce G_2_/M accumulation and NSCs death [25]. Analysis of *Cdkn1a* and *Gadd45a* levels by qRT-PCR 4 h after irradiation revealed that the mRNA expression levels of these genes were significantly increased by increasing doses of radiation (Figure 2C).

**Figure 3:**
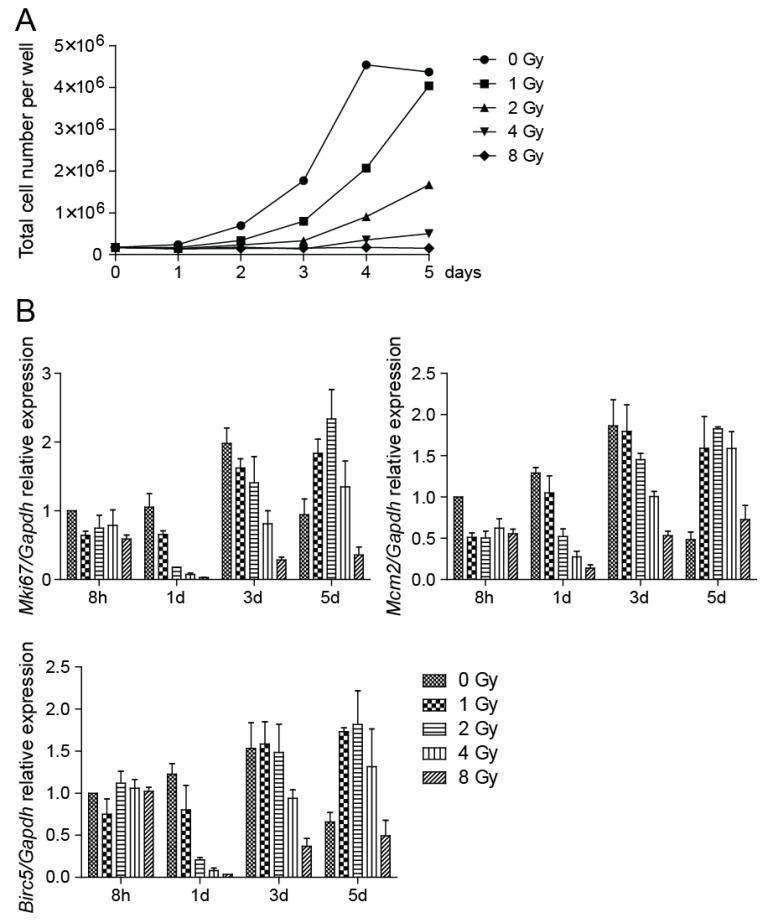
Cell proliferation is impaired by irradiation. (**A**) After irradiation to 0, 1, 2, 4, and 8 Gy doses, cells were counted every day and the total number of cells in control and irradiated plate wells was plotted. (**B**) Quantitative RT-PCR analysis of *Mki67, Mcm2*, and *Birc5* mRNA expression at 8 h and days 1, 3, and 5 after irradiation to 0, 1, 2, 4, and 8 Gy doses. *Gapdh* was used as a reference gene. *Mki67*: *p* < 0.0001, *Mcm2*: *p* < 0.0001, *Birc5*: *p* < 0.0001.

To determine the growth potential following irradiation of NSCs, we cultured cells after exposure to 1, 2, 4, and 8 Gy irradiation and counted the number of cells cultivated in vitro in six-well plates during a five-day period. NSC growth was impaired in a dose-dependent manner. Compared to control cells, which reached a growth plateau on day 4 of cultivation, the growth of cells irradiated to moderate doses was delayed and cells irradiated to 8 Gy doses failed to expand (Figure 3A). We also verified the expression of several proliferation markers, which belong among genes regulated by the p53-DREAM pathway [26]. *Mki67, Mcm2*, and *Birc5* mRNA levels were analyzed by qRT-PCR and we found their expression significantly changed by increasing doses of radiation (Figure 3B). Expression of *Mki67* and *Mcm2* genes decreased at 8 h after the radiation exposure and was dose-dependently reduced when compared to respective controls during the exponential phase of cell growth. The dose-dependent decrease in the expression of the *Birc5* gene, which encodes protein survivin, was first detected at day 1 after the radiation exposure. Survivin is an inhibitor of apoptosis protein [27] that has been reported to bind to and inhibit caspase-3 and -7, which act as terminal effectors in apoptotic protease cascades [28].

**Figure 4:**
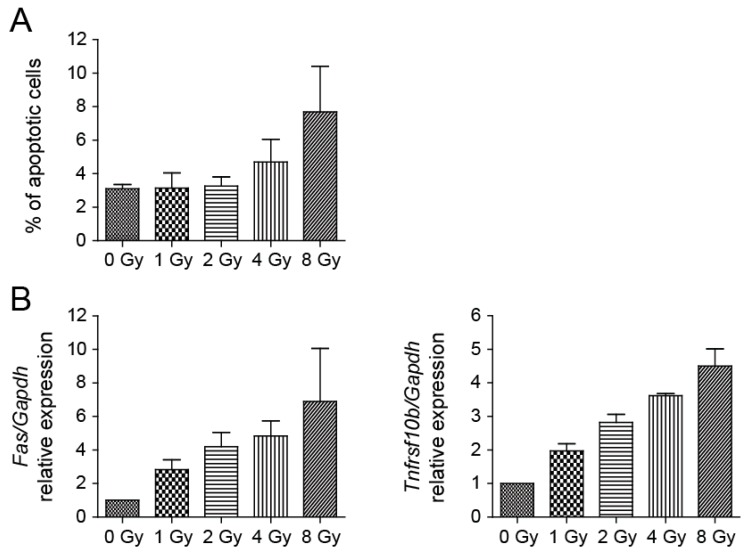
Irradiation of NSCs results in only a mild increase of apoptosis. (**A**) Determination of apoptosis using Caspase-3/7 Assay Kit 1 day after irradiation. Mean values: 0 Gy-3.10, 1 Gy-3.14, 2 Gy-3.26, 4 Gy-4.71, 8 Gy-7.69; *p* = 0.0181. (**B**) *Fas* and *Tnfrsf10b* expression in irradiated and control cells was quantified by qRT-PCR analysis one day after irradiation. *Gapdh* was used as a reference gene. Mean values-*Fas*: 0 Gy-1, 1 Gy-2.83, 2 Gy-4.20, 4 Gy-4.84, 8 Gy-6.90; *p* = 0.0049; -*Tnfrsf10b*: 0 Gy-1, 1 Gy-1.97, 2 Gy-2.82, 4 Gy-3.62, 8 Gy-4.50; *p* < 0.0001.

To analyze the capability of radiation to trigger apoptosis, NSCs were subjected to 1, 2, 4, and 8 Gy doses of irradiation. Analysis of irradiated NSCs using a Caspase-3/7 Assay Kit showed a slightly elevated level of apoptosis in irradiated cells in comparison to control cells. The yield of apoptotic cells in cell culture irradiated to 8 Gy doses was approximately 2.5-fold over the background one day after irradiation (Figure 4A). We also determined the expression levels of a few genes involved in apoptosis and observed their dose-dependent increase. Fas cell surface death receptor (FAS) is a member of the TNF-receptor superfamily, the death domain of which positively regulates apoptosis [29]. The 6.9-fold increase in *Fas* expression by 8 Gy of irradiation appeared to induce apoptosis. Analysis of the expression of the *Tnfrsf10b* gene, which encodes a protein also known as death receptor 5 (DR5) [30], showed an approximate 4.5-fold increase in 8 Gy dose culture (Figure 4B).

Nevertheless, the percentage of apoptotic cells after NSC irradiation was overall low (less than 8% at 8 Gy), indicating that cells respond to irradiation mainly in a different, nonapoptotic way.

### 2.3. Radiation-Induced DNA Damage Promotes NSC Differentiation

We determined the effect of irradiation on gene expression of neural-specific markers in NSCs cultured in vitro. Compared to control cells, irradiated cell cultures exhibited morphological changes upon cultivation in standard growth medium. The most evident change observed in irradiated cells was the formation of elongated cell processes, which indicated the onset of differentiation.

**Figure 5:**
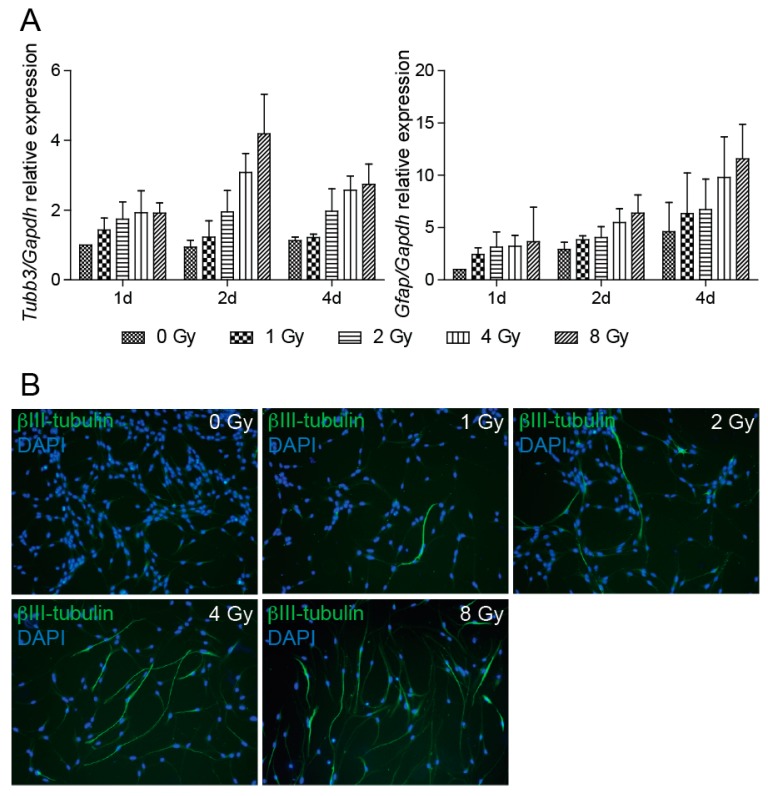
Radiation promotes NSC differentiation. (**A**) Quantitative RT-PCR analysis of mRNA levels of *Tubb3* and *Gfap* genes at 1, 2, and 4 days after irradiation to 0, 1, 2, 4, and 8 Gy doses. *Gapdh* was used as a reference gene. *Tubb3*: *p* < 0.0001, *Gfap*: *p* = 0.0002. (**B**) Representative immunofluorescence images of NSCs 4 days after irradiation to 0, 1, 2, 4, and 8 Gy doses stained with βIII-tubulin antibody (green). DAPI (blue) was used to stain the nuclei. Original magnification 100×.

Quantification of mRNA changes in the expression of βIII-tubulin, which encodes a part of the microtubular complex specific for neurons, clearly illustrates that irradiation results in a dose-dependent increase of βIII-tubulin expression (Figure 5A), which we were able to detect as early as one day after irradiation. The results also demonstrated increased expression of *Gfap* in irradiated cells. GFAP is a type-III intermediate filament protein that is used to identify astrocytes. The qRT-PCR results of βIII-tubulin mRNA expression were confirmed by fluorescent microscopy followed by ImageJ software analysis of cells stained with βIII-tubulin antibody. The analysis showed that, compared to control cells, the number of βIII-tubulin-positive cells is higher in irradiated cells (Figure 5B).

Assessment of the percentage of βIII-tubulin-positive cells in 8 and 4 Gy dose cell cultures showed positivity of 84% and 77% of all cells, respectively. In the 2 Gy dose cell culture, the number of βIII-tubulin-positive cells dropped to 32%; in 1 Gy dose cells, the difference from control cells was not significant. Unfortunately, we were unable to quantify cells stained by GFAP antibody due to very low protein expression, which did not allow counting of GFAP-positive cells.

Taken together, we demonstrated the potential of irradiated NSCs cultivated in vitro to differentiate into neurons.

## 3. Discussions

In this study, we examined the effects of irradiation on NSCs derived from the mouse brain and grown in vitro. First, we analyzed the characteristics of NSCs, as published results indicate that different subpopulations of neural stem cells may respond differently to radiation exposure [31]. Purification of V-SZV NSCs from the mouse brain revealed that except for the major types of neural stem cells (qNSCs, aNSCs, and NPCs), additional subpopulations in intermediate states exist. For example, pseudotemporal ordering, based on single-cell transcription profiling data, revealed three subpopulations of aNSCs, which exhibit differential expression of specific genes [22]. Under the specified culture conditions, we found that NSCs cultured in vitro express high levels of stemness markers nestin and *Sox2*, proliferation markers *Mki67* and *Mcm2,* and high levels of the *Egfr* marker. We detected lower expression of astrocytic marker *Gfap* and were unable to detect the expression of the *Dcx* gene. Using immunocytochemical analysis we determined the percentage of Ki-67-negative cells to be 22%. According to single-cell transcription profiling data published by Dulken et al. [22] and summarized by Obernier et al. [12], the expresion of the *Egfr* marker is specific for aNSC and NPC subpopulations, while expression of the *Dcx* gene is characteristic of NPCs and neuroblasts. Analysis of NSCs purified from the mouse brain also revealed that the percentage of Ki-67-negative cells is about 23% in the aNSC population and about 14% in the NPC population [22]. Taken together, comparing our results with immunocytochemical and single-cell transcription profiling data mentioned above, we concluded that in our experiments, most cultured cells displayed features of the aNSC state.

To assess the DNA damage after NSC irradiation we employed immunofluorescence staining of γ-H2AX foci. We showed that compared to sham-irradiated control, cultures of NSCs showed bright γ-H2AX foci after irradiation, the numbers of which increased by increasing doses of radiation. It is known that many γ-H2AX foci could be found in the cell nuclei during M phase of the cell cycle, even though without any DNA damage [32]. Nevertheless, we found the percentage of cells with a high number of γ-H2AX foci in control NSCs is relatively low (less than 2%). Radiation exposure leads to diverse outcomes across different tissues but is at least partially attributable to the status of tumor suppressor p53, and activation of its transcriptional targets [33]. We determined the transcriptional activity of p53 targets *Cdkn1a* and *Gadd45a* and found that the mRNA expression levels of these genes were increased by increasing doses of radiation, which indicates NSC growth impairment. Cell cultivation after irradiation then confirmed that the NSC growth potential is impaired in a dose-dependent manner. While control cells reached a growth plateau on day 4 of cultivation and no longer grew to be completely confluent, cells irradiated to 8 Gy doses failed to expand in the course of the entire monitored period. The verification of the expression levels of genes associated with cell proliferation shows dose-dependently reduced expression when compared to respective controls during the exponential phase of cell growth. Obviously, when control cells reached the growth plateau and no longer proliferated, the expression of the analyzed proliferation markers was reduced. A decrease in the expression of proliferation marker *Mki67* has been previously described in in vivo experiments where the reduction in the number of proliferative Ki-67-positive cells in the V-SVZ niche of adult mouse brain was confirmed several days post-irradiation [31]. We also analyzed the capability of radiation to trigger apoptosis in NSCs and found that the percentage of apoptotic cells after NSC irradiation is low, which indicates that cells respond to irradiation in another, nonapoptotic way. In vivo, ionizing radiation, by inducing acute apoptosis in dividing cells, reduces the pool of mitotic NSCs and consequently reduces generation of new neurons [15]. Until recently, the general consensus was that irradiation provokes apoptosis of proliferating cells, including aNSCs and NPCs [16,17]. Nevertheless, Barazzuol et al. [31] showed that sensitivity to apoptosis rather reflects the cell type than the proliferative status of NSCs and characterized two novel responses to radiation. Proliferation arrest and progenitor marker loss represent additional radiation-induced responses that are distinct from apoptosis. Moreover, they showed that radiation exposure promotes differentiation of NSCs in the adult V-SVZ niche.

During the cultivation of NSCs in standard growth medium, we noticed that irradiated cell cultures exhibited morphological changes, mainly formation of elongated cell processes. We confirmed the effect of irradiation on enhanced gene expression of neural-specific markers, validated the increase of the percentage of βIII-tubulin-positive cells, and thus demonstrated the potential of irradiated NSCs to differentiate into neurons. Growing evidence suggests that terminal differentiation is the stem cell way to react to fatal DNA damage. The induction of differentiation in the damaged NSCs may eliminate potential hazardous cells and thereby clear them from the system. In 2005, Lin et al. [34] reported enhanced differentiation of embryonic stem cells mediated by p53 in response to UV or doxorubicin treatment. Their findings indicated that apoptosis is not efficient in ESCs after DNA damage and proposed an alternative mechanism to maintain genetic stability by inducing the differentiation program. In hematopoietic stem cells (HSCs), GADD45A induces a strong differentiation program via inhibition of the p38 MAPK signaling pathway. GADD45A is known to coordinate cellular stress responses and, induced by p53, arrests the cell cycle at the G_2_/M phase. The observed terminal differentiation of damaged HSCs seems to be a prominent function of GADD45A in the hematopoietic system that prevents their fatal transformation and maintains genomic stability of cells if they harbor excess DNA damage [35]. Our results show that most cells cultivated in in vitro conditions demonstrated features of proliferative aNSCs; nevertheless, they did not undergo apoptosis after irradiation. They preferred to cease proliferation and start a differentiation program. Distinct responses to ionizing radiation between NSCs and neural progenitor cells were shown in vivo [31]. In vivo analysis provides evidence that progenitor cells, but not qNSCs and aNSCs, undergo apoptosis after 2 Gy irradiation, and thus the sensitivity to apoptosis rather seems to be a feature of the cell types than a direct consequence of proliferation per se. DNA damage-induced differentiation was proposed to be a response mechanism to irradiation in qNSCs and aNSCs, which in these cell types may circumvent the fatal consequences of cumulative DNA damage. Nevertheless, the induction of premature differentiation in the damaged, irradiated NSCs may lead to a considerable cell loss. In neurogenic zone of the adult mouse brain, qNSCs give rise to aNSCs, which finally differentiate into neuroblasts [10,11,12]. At moderate doses of irradiation, proliferation in the neurogenic niches restarts a few days after exposure by recruiting qNSCs [16,17]. However, at higher doses, endothelial cells that line the interior surface of blood vessels in the stem cell niche increase production of TGF-β1, which supports qNSC dormancy [20] and leads to qNSCs’ inability to replace the cells lost.

The main aim of clinical brain tumor radiotherapy is to destroy cancer cells while causing minimal damage to the surrounding healthy tissues. As irradiation to neurogenic niches causes a decline in cognitive functions [36], there is a growing effort to spare cells in this region. Limitation of the delivered dose into neurogenic zones can be achieved using the modern radiotherapy techniques such as intensity-modulated radiotherapy, where the dose is delivered using nonuniform beams. This approach enables physicians to achieve delivery of the full treatment dose only within the designated treatment volume, with maximal sparing of the healthy tissues [37]. Much effort is nowadays also dedicated to finding pre-irradiation treatments that may prevent the negative effects of radiation on NSCs. This is especially important in the course of irradiation of the juvenile brain, as the neonatal progenitor cells have diminished ability to undergo proliferative arrest and, as a consequence, are more at risk to become carcinogenic [31]. As a pretreatment option, lithium has been explored, which increases proliferation of NSCs and rescues radiation-induced cell cycle arrest [38]. Further, it has been demonstrated that melatonin, a regulator of circadian rhythm, decreases apoptosis and upregulates neural stem cell markers in the V-SZV zone of irradiated rats [39]. In vivo radiation therapy reduces adult neurogenesis not only by reducing the pool of mitotic neural stem cells, but also due to the changes within the NSC microenvironment. Experiments demonstrated that transplanted nonirradiated precursors cells are unable to differentiate in an irradiated hippocampus [19]. This shows that even when qNSCs survive irradiation and thus are potentially able to activate and reconstitute neurogenesis, such process may be suppressed by inflammation and vascular damage in the stem cell niche. Cultures of primary NSCs have been used to study NSCs in vitro, although it is still debated whether these cells are good models for in vivo NSCs [40]. Neurosphere- and monolayer-based protocols have allowed isolation of developmental stage-specific NSC populations and generation of pure cultures of NSCs that can be stably maintained over passages. In contrast, NSCs expanded in vitro can undergo deregulation of some of their original properties due to the absence of the neurogenic niche. Although single-cell transcriptomics helps to understand differences between in vivo and in vitro situations and shows that in vitro cultured NSCs resemble in vivo aNSCs [22], one has to keep in mind that the impact of the stem cell niche may be substantial.

## 4. Materials and Methods

### 4.1. Cell Isolation and Cultivation

Generation and housing of C57BL/6 mice were carried out in compliance with the Directive 2010/63/EU on the protection of animals used for scientific purposes and national and institutional guidelines. The V-SVZ zone was dissected from brains of adult (8-week-old) mice as described previously [41]. Neural stem cells were derived from the V-SVZ zone according to the protocol established by Walker et.al. [42]. Cells were cultured in a growth medium prepared by combining DMEM/F12 medium (Sigma, St. Louis, MO, USA) containing N2 supplement (Gibco, Waltman, MA, USA) and Neurobasal medium (Gibco) containing B27 supplement (Gibco) and 2 mM L-glutamine (Gibco). The final medium was supplemented with 100 U penicillin/mL and 10 μg streptomycin/mL (Gibco), BSA (25 μg/mL, Gibco), insulin (12.5 μg/mL, Sigma), apo-transferrin (50 μg/mL, Sigma), FGF-2 (10 ng/mL, R&D Systems, Minneapolis, MN, USA) and EGF (10 ng/mL, R&D Systems). Cells were passaged every 3rd day using 0.05% trypsin/EDTA and reseeded into poly-ornithine/laminin-coated tissue culture flasks at a density of 0.8 × 10^4^/cm^2^.

The study has been approved by the Animal Facility Committee of the Institute of Molecular Genetics, CAS; code 8/2016, approved on 8 January 2016.

### 4.2. RNA Extraction and Real-Time qRT-PCR

Total RNA was isolated from cells using a PureLink RNA Mini Kit (Ambion, Waltham, MA, USA) according to the manufacturer’s protocol. Two hundred nanograms of total RNA was reverse transcribed using random hexamer primers (Invitrogen, Waltham, MA, USA) and M-MLV Reverse Transcriptase (Promega, Madison, WI, USA). cDNAs were amplified by the LightCycler^®^ 480 system (Roche, Basel, Switzerland) using the SYBR Green I Master mix (Roche). All reactions were performed in triplicates and all mRNA levels were normalized to Gapdh mRNA.

### 4.3. Primers

The following primers were used:

Gapdh: 5′-TGTGTCCGTCGTGGATCTGAC-3′ and 5′-TTGCTGTTGAAGTCGCAGGAG-3′,

Nes: 5′-AGGCTGAGAACTCTCGCTTGC-3′ and 5′ -GGTGCTGGTCCTCTGGTATCC-3′,

Sox2: 5′-TACCTCTTCCTCCCACTCCA-3′ and 5′-CTGGGCCATGTGCAGTCTA-3′,

Egfr: 5′-AGGCCGTGAACCACGTCTGC-3′ and 5′-CACGCACTCCCTGCCTCTGC3′,

Mcm2: 5′-AAGGCTGGCATCGTTACCTC-3′ and 5′-GGTCAGTGAAGGGTCGTAGC3′,

Mki67: 5′-GCCTCCTAATACACCACTGA-3′ and 5′-CCGTTCCTTGATGATTGTCTT3′,

Gfap: 5′-TGAGGCAGAAGCTCCAAGA-3 ′ and 5′-CCAGGGTGGCTTCATCTGC-3′,

Prom1: 5′-GCCTCTACCCTGGAAGCAAA-3′ and 5′-GATGCTGGTGGATGGCTCTT3′,

Dcx: 5′-GAACAAGGACTTTGTGCGCC-3′ and 5′-CAGTCAGGACCTGCTCGAAA3′,

Tubb3: 5′-TGGACAGTGTTCGGTCTGG-3′ and 5′-CCCTCCGTATAGTGCCCTTTG-3′,

Cdkn1a: 5′-GTCTGAGCGGCCTGAAGAT-3′ and 5′-TCTGCGCTTGGAGTGATAGA-3′,

Gadd45a: 5′-CTGCTGCTACTGGAGAACGA-3′ and 5′-GGATCCTTCCATTGTGATGAA-3′,

Birc5: 5′-TACCGAGAACGAGCCTGATT-3′ and 5′-CAGGGGAGTGCTTTCTATGC-3′,

Fas: 5′-GTGAACATGGAACCCTTGAGCC-3′ and 5′-TGGTCAACAACCATAGGCGA-3′,

Tnfrsf10b: 5′-GTTGCTGCTTGCTGTGCTAC-3′ and 5′-GGTCCTCTTGATGGGCTCTC-3′

### 4.4. Cell Irradiation

Cells were irradiated at room temperature with single doses of 0, 1, 2, 4, and 8 Gy. One day before irradiation, cells were plated onto poly-ornithine/laminin-coated six-well plates at a density of 1.2 × 10^4^/cm^2^ and cultured in the growth medium. Irradiation was performed with a photon beam produced in a 4 MV Varian Clinac 600C/D DBX linear accelerator. The dose rate was 200 MU/min, where 100 MU corresponds to a dose of 1 Gy in depth of 10 cm in water. The gantry angle was set to 180°. Between the cells and a treatment table, two plastic plates were present to avoid a build-up region. The required amount of MU was calculated for all of the doses using the Elekta’s treatment planning system Monaco 5.11.02, where the real geometry was simulated with Monte Carlo algorithm. The medium was replaced by fresh growth medium immediately after irradiation in both, control and irradiated cells.

### 4.5. Immunofluorescence and Imaging

To detect proteins via immunofluorescence, cells were grown on glass coverslips, fixed in 4% paraformaldehyde, permeabilized with 0.1% Triton X-100, and blocked in a mixture of 10% NGS (Jackson ImmunoResearch, Cambridge, UK) and 5% BSA (Sigma) before incubation with a primary antibody. The following primary antibodies were used: mouse anti-nestin (1:500, Millipore, Burlington, MA, USA), mouse anti-SOX2 (1:500, Sigma), rabbit anti-Ki-67 (1:400, DBS, Pleasanton, CA, USA), mouse anti-phospho histone H2A.X (γH2AX, 1:300, Millipore) and mouse anti-β III-tubulin (1:1000, R&D Systems). Nestin, SOX2, γH2AX and β III-tubulin staining was visualized by goat anti-mouse IgG Alexa Fluor 488 secondary antibody (Invitrogen); Ki-67 staining was visualized by goat anti-rabbit IgG Alexa Fluor 488 secondary antibody (Invitrogen). To visualize nuclei, cells were stained with DAPI (Sigma). Representative pictures were acquired using a Leica DM6000 microscope. The percentage of Ki-67-positive cells, number of γH2AX foci per nuclei, and percentage of βIII-tubulin-positive cells were counted manually using the ImageJ software (version 1.48, NIH, USA; Cell Counter plugin). Multiple fields (between 300–500 cells) per each staining in four independent experiments were counted.

### 4.6. Analysis of Cell Growth and Apoptosis

The cell growth curve was determined by counting the cell number on particular days using Muse^®^ Cell Analyzer (Millipore) and Muse^®^ Count &Viability Assay Kit (Millipore). The level of apoptosis was detected 24 h after irradiation. Muse^®^ Caspase-3/7 Assay Kit (Millipore) was used according to the manufacturer’s protocol and stained cells were measured by Muse^®^ Cell Analyzer (Millipore).

### 4.7. Statistical Analysis

The results obtained in the qRT-PCR and ImageJ analysis were processed using GraphPad Prism software version 6 (San Diego, CA, USA). All data represent the mean of four biological replicates with error bars indicating the standard deviation of the data. To determine the statistical significance of the results, the ANOVA test was used.

## 5. Conclusions

In the current study, we investigated the effects of irradiation on NSCs derived from mouse brains and grown in vitro. Our findings describe the increased transcriptional activity of p53 targets, including *Gadd45a*, and proliferative arrest after irradiation. Moreover, we show that most cells do not undergo apoptosis after irradiation but rather cease proliferation and start a differentiation program. Induction of differentiation and the demonstrated potential of irradiated cells to differentiate into neurons may represent a mechanism through which damaged NSCs circumvent the consequences of cumulative DNA damage.

## References

[1] Cancer Today. http://gco.iarc.fr/today/home.

[2] Mahase S.S., Navrazhina K., Schwartz T.H., Parashar B., Wernicke A.G. (2019). Intraoperative brachytherapy for resected brain metastases. Brachytherapy.

[3] Chang E.L., Wefel J.S., Hess K.R., Allen P.K., Lang F.F., Kornguth D.G., Arbuckle R.B., Swint J.M., Shiu A.S., Maor M.H. (2009). Neurocognition in patients with brain metastases treated with radiosurgery or radiosurgery plus whole-brain irradiation: A randomised controlled trial. Lancet Oncol..

[4] Deangelis L.M., Delattre J.Y., Posner J.B. (1989). Radiation-Induced Dementia in Patients Cured of Brain Metastases. Neurology.

[5] Mulhern R.K., Merchant T.E., Gajjar A., Reddick W.E., Kun L.E. (2004). Late neurocognitive sequelae in survivors of brain tumours in childhood. Lancet Oncol..

[6] Gibson E., Monje M. (2012). Effect of cancer therapy on neural stem cells: Implications for cognitive function. Curr. Opin. Oncol..

[7] Kut C., Redmond K.J. (2014). New Considerations in Radiation Treatment, Planning for Brain Tumors: Neural Progenitor Cell-Containing Niches. Semin. Radiat. Oncol..

[8] Doetsch F., Garcia-Verdugo J.M., Alvarez-Buylla A. (1997). Cellular composition and three-dimensional organization of the subventricular germinal zone in the adult mammalian brain. J. Neurosci..

[9] Kuhn H.G., Dickinson-Anson H., Gage F.H. (1996). Neurogenesis in the dentate gyrus of the adult rat: Age-related decrease of neuronal progenitor proliferation. J. Neurosci..

[10] Llorens-Bobadilla E., Zhao S., Baser A., Saiz-Castro G., Zwadlo K., Martin-Villalba A. (2015). Single-Cell Transcriptomics Reveals a Population of Dormant Neural Stem Cells that Become Activated upon Brain Injury. Cell Stem Cell.

[11] Codega P., Silva-Vargas V., Paul A., Maldonado-Soto A.R., Deleo A.M., Pastrana E., Doetsch F. (2014). Prospective identification and purification of quiescent adult neural stem cells from their in vivo niche. Neuron.

[12] Obernier K., Alvarez-Buylla A. (2019). Neural stem cells: Origin, heterogeneity and regulation in the adult mammalian brain. Development.

[13] Yu H. (2012). Typical cell signaling response to ionizing radiation: DNA damage and extranuclear damage. Chin. J. Cancer Res..

[14] Sancar A., Lindsey-Boltz L.A., Unsal-Kacmaz K., Linn S. (2004). Molecular mechanisms of mammalian DNA repair and the DNA damage checkpoints. Annu. Rev. Biochem..

[15] Mizumatsu S., Monje M.L., Morhardt D.R., Rola R., Palmer T.D., Fike J.R. (2003). Extreme sensitivity of adult neurogenesis to low doses of X-irradiation. Cancer Res..

[16] Bellinzona M., Gobbel G.T., Shinohara C., Fike J.R. (1996). Apoptosis is induced in the subependyma of young adult rats by ionizing irradiation. Neurosci. Lett..

[17] Daynac M., Chicheportiche A., Pineda J.R., Gauthier L.R., Boussin F.D., Mouthon M.A. (2013). Quiescent neural stem cells exit dormancy upon alteration of GABA(A)R signaling following radiation damage. Stem Cell Res..

[18] Lazarini F., Mouthon M.A., Gheusi G., de Chaumont F., Olivo-Marin J.C., Lamarque S., Abrous D.N., Boussin F.D., Lledo P.M. (2009). Cellular and Behavioral Effects of Cranial Irradiation of the Subventricular Zone in Adult Mice. PLoS ONE.

[19] Monje M.L., Mizumatsu S., Fike J.R., Palmer T.D. (2002). Irradiation induces neural precursor-cell dysfunction. Nat. Med..

[20] Pineda J.R., Daynac M., Chicheportiche A., Cebrian-Silla A., Felice K.S., Garcia-Verdugo J.M., Boussin F.D., Mouthon M.A. (2013). Vascular-derived TGF- increases in the stem cell niche and perturbs neurogenesis during aging and following irradiation in the adult mouse brain. EMBO Mol. Med..

[21] Schneider L., Pellegatta S., Favaro R., Pisati F., Roncaglia P., Testa G., Nicolis S.K., Finocchiaro G., di Fagagna F.D. (2013). DNA Damage in Mammalian Neural Stem Cells Leads to Astrocytic Differentiation Mediated by BMP2 Signaling through JAK-STAT. Stem Cell Rep..

[22] Dulken B.W., Leeman D.S., Boutet S.C., Hebestreit K., Brunet A. (2017). Single-Cell Transcriptomic Analysis Defines Heterogeneity and Transcriptional Dynamics in the Adult Neural Stem Cell Lineage. Cell Rep..

[23] Meletis K., Wirta V., Hede S.M., Nister M., Lundeberg J., Frisen J. (2006). P53 suppresses the self-renewal of adult neural stem cells. Development.

[24] El-Deiry W.S., Tokino T., Velculescu V.E., Levy D.B., Parsons R., Trent J.M., Lin D., Mercer W.E., Kinzler K.W., Vogelstein B. (1993). WAF1, a potential mediator of p53 tumor suppression. Cell.

[25] Galvin K.E., Ye H., Erstad D.J., Feddersen R., Wetmore C. (2008). Gli1 induces G2/M arrest and apoptosis in hippocampal but not tumor-derived neural stem cells. Stem Cells.

[26] Engeland K. (2018). Cell cycle arrest through indirect transcriptional repression by p53: I have a DREAM. Cell Death Differ..

[27] Ambrosini G., Adida C., Altieri D.C. (1997). A novel anti-apoptosis gene, survivin, expressed in cancer and lymphoma. Nat. Med..

[28] Shin S., Sung B.J., Cho Y.S., Kim H.J., Ha N.C., Hwang J.I., Chung C.W., Jung Y.K., Oh B.H. (2001). An anti-apoptotic protein human survivin is a direct inhibitor of caspase-3 and-7. Biochemistry.

[29] Kuwabara M., Takahashi K., Inanami O. (2003). Induction of apoptosis through the activation of SAPK/JNK followed by the expression of death receptor Fas in X-irradiated cells. J. Radiat. Res..

[30] Sheridan J.P., Marsters S.A., Pitti R.M., Gurney A., Skubatch M., Baldwin D., Ramakrishnan L., Gray C.L., Baker K., Wood W.I. (1997). Control of TRAIL-induced apoptosis by a family of signaling and decoy receptors. Science.

[31] Barazzuol L., Ju L.M., Jeggo P.A. (2017). A coordinated DNA damage response promotes adult quiescent neural stem cell activation. PLoS Biol..

[32] Ichijima Y., Sakasai R., Okita N., Asahina K., Mizutani S., Teraoka H. (2005). Phosphorylation of histone H2AX at M phase in human cells without DNA damage response. Biochem. Biophys. Res. Commun..

[33] Lee C.L., Blum J.M., Kirsch D.G. (2013). Role of p53 in regulating tissue response to radiation by mechanisms independent of apoptosis. Transl. Cancer Res..

[34] Lin T.X., Chao C., Saito S., Mazur S.J., Murphy M.E., Appella E., Xu Y. (2005). P53 induces differentiation of mouse embryonic stem cells by suppressing Nanog expression. Nat. Cell Biol..

[35] Wingert S., Thalheimer F.B., Haetscher N., Rehage M., Schroeder T., Rieger M.A. (2016). DNA-Damage Response Gene GADD45A Induces Differentiation in Hematopoietic Stem Cells Without Inhibiting Cell Cycle or Survival. Stem Cells.

[36] Pereira Dias G., Hollywood R., Bevilaqua M.C., da Luz A.C., Hindges R., Nardi A.E., Thuret S. (2014). Consequences of cancer treatments on adult hippocampal neurogenesis: Implications for cognitive function and depressive symptoms. Neuro-Oncology.

[37] Hermanto U., Frija E.K., Lii M.F.J., Chang E.L., Mahajan A., Woo S.Y. (2007). Intensity-modulated radiotherapy (IMRT) and conventional three-dimensional conformal radiotherapy for high-grade gliomas: Does IMRT increase the integral dose to normal brain?. Int. J. Radiat. Oncol..

[38] Zanni G., Di Martino E., Omelyanenko A., Andang M., Delle U., Elmroth K., Blomgren K. (2015). Lithium increases proliferation of hippocampal neural stem/progenitor cells and rescues irradiation-induced cell cycle arrest in vitro. Oncotarget.

[39] Naseri S., Moghahi S.M.H.N., Mokhtari T., Roghani M., Shirazi A.R., Malek F., Rastegar T. (2017). Radio-Protective Effects of Melatonin on Subventricular Zone in Irradiated Rat: Decrease in Apoptosis and Upregulation of Nestin. J. Mol. Neurosci..

[40] Conti L., Cattaneo E. (2010). Neural stem cell systems: Physiological players or in vitro entities?. Nat. Rev. Neurosci..

[41] Azari H., Rahman M., Sharififar S., Reynolds B.A. (2010). Isolation and expansion of the adult mouse neural stem cells using the neurosphere assay. J. Vis. Exp..

[42] Walker T.L., Kempermann G. (2014). One mouse, two cultures: Isolation and culture of adult neural stem cells from the two neurogenic zones of individual mice. J. Vis. Exp..

